# Phyllosilicates as protective habitats of filamentous cyanobacteria *Leptolyngbya* against ultraviolet radiation

**DOI:** 10.1371/journal.pone.0219616

**Published:** 2019-07-11

**Authors:** Alex Kugler, Hailiang Dong

**Affiliations:** 1 Department of Geology and Environmental Earth Sciences, Miami University, Oxford, OH, United States of America; 2 State Key Laboratory of Biogeology and Environmental Geology, China University of Geosciences, Beijing, China; CEA-Saclay, FRANCE

## Abstract

Phototrophic cyanobacteria are limited in growth locations by their need for visible light and must also cope with intermittent ultraviolet radiation (UVR), especially in extreme environments such as deserts and on early Earth. One survival method for cyanobacteria is growing endolithically within minerals such as micas, gypsum, and quartz minerals. However, the capability of different mica minerals to protect cyanobacteria from UVR, while at the same time allowing transmission of photosynthetically active radiation (PAR), has only been minimally examined. In this study, we performed laboratory incubation experiments to demonstrate that a model filamentous cyanobacterium, *Leptolyngbya* sp., can colonize micas, such as muscovite, phlogopite, and biotite. After inoculation experiments confirmed that these cyanobacteria grew between the sheets of mica, *Leptolyngbya* sp. colonies were exposed to UVB and UVC for up to 24 hrs, and the level of survival was determined using chlorophyll a and carotenoid assays. Of the three micas investigated, muscovite, being an Fe-poor and Al-rich mica, provided the least attenuation of UVR, however it transmitted the most visible light. Fe-rich biotite provided the best UVR shielding. Phlogopite, apparently because of its intermediate amount of Fe, showed the greatest ability to shield UVR while still transmitting an adequate amount of visible light, making it the ideal habitat for the cyanobacterium. Upon exposure to UVR, significant shifts in several important fatty acids of the cyanobacterium were detected such as linolenic acid and oleic acid, 18:3ω3 and 18:1ω9c, respectively. These cellular changes are interpreted to be a consequence of UVR and other accessory stress (such as O_3_).

## Introduction

Cyanobacteria are found in a number of terrestrial environments [1]. In deserts and on early earth, intense ultraviolet radiation (UVR, 280–400 nm in wavelength) can cause various damages to these organisms, such as protein and DNA damage [2], and pigment degradation [3]. In particular, chlorophyll *a* and carotenoids, two major pigments in cyanobacteria, are degraded by UVR but at different rates, with carotenoids generally more resistant than chlorophyll *a* [4]. To counteract these damaging UVR, cyanobacteria have developed multiple defense mechanisms [5–7]. First, these organisms use UV-screening pigments such as mycosporine-like amino acids and scytonemin. These special amino acids are a common group of transparent, UVR absorbing intracellular compounds [4, 8]. Scytonemin is a yellowish brown pigment contained in certain extracellular sheaths of cyanobacteria, and is capable of absorbing radiation in the UVA range [9]. Second, a major class of cyanobacterial pigments, carotenoids, is known for their antioxidant activity and can remove reactive oxygen species in oxidizing environments, and thus prevents lipid peroxidation [10, 11]. Third, there is some indication that cyanobacteria may be able to alter their lipid profiles [12]. For example, shifts in lipid profiles have been observed for microbial communities in global deserts [13]. Considering lipid compositions are sensitive to other environmental stresses such as temperature [12], salinity [14], metal contamination [15], starvation [16], and light condition [17], it is reasonable to expect change of lipid composition in response to UVR, but such studies have not been performed. Lastly, certain cyanobacterial species can repair damaged DNA and/or turn over proteins to reverse the UVR-induced damage [18].

There are physical strategies that some cyanobacteria, mostly filamentous organisms such as *Chroococcidiopsis* and *Leptolyngbya* [19, 20], could use to survive UV-intense environments. For example, in desert environments, where UVR can reach as high as 840 GJ km^-2^ year^-1^ [21], cyanobacteria can reside inside various rocks and minerals for protection, e.g., endolithic growth [22]. In Antarctic and Atacama deserts, cyanobacteria are usually found growing within a variety of minerals and rocks [23–26]. Translucent sedimentary rocks [27, 28], siliceous rocks of geothermal origin [29], gypsum in soils and evaporate deposits [30], and carbonate rocks [26, 31–33] are common habitats for those organisms. These rocks are sufficiently translucent to transmit the light necessary for photosynthesis [34], but sufficiently opaque to block or attenuate UVR. In carbonate rocks, euendolithic cyanobacteria can specialize into particular rock types, with the cationic mineral component being as the determinant in this specialization [33]. Recently, phototrophic communities have also been found in various darker-hued rocks such as crystalline basalt, obsidian, and biotite, however, the depth at which cyanobacteria can colonize is fairly shallow due to their requirement for adequate visible light penetration [35]. In addition to protecting against UVR, these rocks and minerals also provide protection against excessive photosynthetically active radiation [36], maintain a stable temperature [37], serve as a source for trace and major nutrients [38], increase availability of moisture [39], and reduce habitat loss by erosive winds [40].

Studies have shown that Fe-bearing minerals are efficient in protecting microorganisms against UVR [41, 42], because iron is an efficient absorber of such radiation [43]. Indeed, biotite, one particular member of the mica family, has been studied for its role as a potential habitat for cyanobacteria, because this mineral contains Fe in the structure. Biotite can serve as a favorable substrate for endolithic colonization [44] by attenuating UVR but still allowing transmission of some visible light for photosynthesis [45]. However, if the Fe content in a mineral is too high, it may block photosynthetically active radiation (PAR), which would create an unfavorable environment for phototrophs. Therefore, a fine balance between adequate attenuation of UVR and sufficient transmission of PAR must be achieved for phototrophic cyanobacteria to find a best niche in harsh environments. Despite abundant presence of phyllosilicates in desert environments[46–48], the habitability potential of these minerals remains unclear.

The objective of this study was therefore to investigate the effectiveness of mica minerals of different Fe contents in protecting cyanobacteria against UVR. We aim to see if euendolithic cyanobacteria can grow into mica minerals, and if this growth habitat offers protection again UVR in harsh environments. We hypothesize that UVR attenuation and PAR transmittance is a function of the structural Fe content in mica minerals. To achieve this objective and test the hypothesis, we performed survival experiments of a model cyanobacterium in the presence of mica minerals of different Fe contents, where filamentous cyanobacteria were irradiated under UVR with the protection of three mica minerals. Following irradiation, biofilms were imaged to visualize cell damage. Biomass, chlorophyll *a*, and carotenoid pigments were assayed to quantitatively determine the level of protection by various mica minerals. This study suggests that the level of protection is indeed related to the structural Fe content, and of the three micas investigated, phlogopite is most effective in protecting cyanobacteria from UVR, while still allowing transmission of an adequate amount of visible light for photosynthesis. The results of this study have ecological relevance to desert environment and early Earth where cyanobacteria and phyllosilicates may co-exist [46].

## Materials and methods

### Chemicals

All chemicals were of reagent grade unless otherwise specified, and ultra-pure water (18.2 MΩ) was supplied by a Barnstead Thermofisher purification system. HPLC grade methanol from Sigma was used for pigment extraction. All materials, unless otherwise specified, were supplied by Fisher Scientific.

### Minerals

Three mica minerals were selected for their different Fe contents and light transmittance. Muscovite (KAl_2_(Si_3_Al)O_10_(OH)_2_) is transparent and should be effective in transmitting light. Biotite ((K(Mg, Fe)_3_AlSi_3_O_10_(OH)_2_), due to its darker hue, should be opaque to light. Phlogopite, (ideally KMg_3_(AlSi_3_O_10_)(OH)_2_), is the magnesium-rich, Fe poor end member of the phlogopite-biotite solid solution series, and is expected to be between muscovite and biotite with respect to light transmittance. A small amount of Fe may replace Mg in the octahedral site of the phlogopite structure. Samples of muscovite (Madras, India) and biotite (Bancroft, Ontario) were obtained from Wards Scientific. Samples of phlogopite (Bamble, Telemark, Norway) were obtained from the Geology Superstore (www.thegeologysuperstore.com).

Prior to use, all minerals were sterilized in an autoclave at 121°C and 15 psi for one hour so that there would not be interference from ambient microorganisms. At this temperature and pressure chemical changes do not occur [49, 50]. Our control experiments showed that the autoclaving did not change the light transmittance properties of the minerals (visible light at 450 nm and 680 nm and UVR at 302 nm). For the protection experiments the exact mineral thicknesses were determined using calipers. Ten distinct locations on a single mineral were measured, and the averaged thicknesses were reported.

### Cyanobacterium

*Leptolyngbya* sp., ATCC 29170 [51], a filamentous cyanobacterium isolated from Maha Oya, Sri Lanka, was purchased from the American Type Culture Collection. It is routinely cultured in BG-11 type medium [51]. Two different strains of *Leptolyngbya* sp. have been shown to infiltrate biotite flakes in a previous study [45]. Strain 29170 was cultured in BG-11 medium at room temperature under a 5000 K daylight growth lamp (Omni directional GP19 daylight bulb), approximately 22 μmol m^-2^ s^-1^ in light intensity, set at 18/6 hour intervals of light and darkness for optimal growth. Cells were grown within a laminar flow hood, Labconco Logic Plus Biosafety Cabinet, in borosilicate Erlenmeyer flasks capped with cotton wool bungs on a horizontal shaker plate at 50 rpm. Cell growth was monitored with optical density at 600 nm and log phase cells were transferred to petri dishes for subsequent experiments. The optical density at 600 nm was chosen due to its ease of generating and measuring this wavelength within the visible spectrum [52, 53]. In case cells formed clumps, cell suspensions were homogenized via vortexting before optical density measurement.

### Mica infiltration experiments

**Fig 1:**
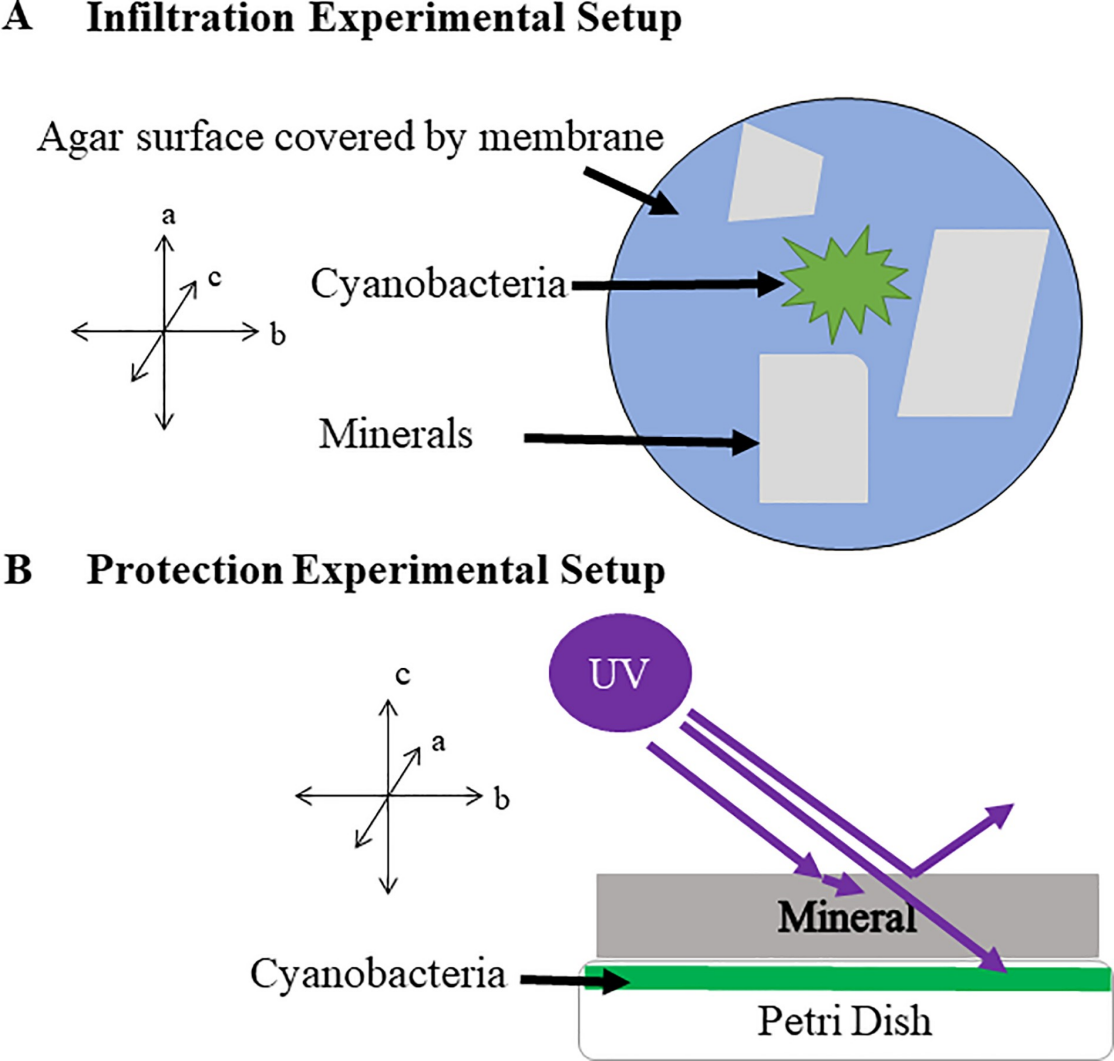
Experimental setup schematics. A). Infiltration experimental setup. *Leptolyngbya* cells were inoculated on the surface of a dialysis membrane covered agar, and three mineral flakes, each at approximately 1 cm (length) x 1 cm (width) x 0.25 (thickness) cm in size, were placed nearby. Cells were allowed to grow into the mica sheets for 2 weeks. B). Protection experimental setup. *Leptolyngbya* cells were inoculated on the surface of membrane-covered agar surface in a petri dish without mineral flakes. After growth for two weeks, the grown biofilm in petri dish was placed under a mica mineral flake and was exposed to UVR.

Petri dishes containing BG-11 medium in 1.5% agar (Fisher BioReagents, Cat.# BP1423-500) were made in a UV sterilized laminar flow hood. A dialysis membrane (pore size 12–14,000 Da, Spectrum Labs) was placed on the cooled agar surface to prevent cyanobacterial growth into the agar and to encourage growth into mica flakes. Mica minerals were cleaved into thin sheets, approximately 1 cm (length) x 1 cm (width) x 0.25 (thickness) cm in size. Within each petri dish, three flakes of a given mineral were placed on top of the dialysis membrane, and then inoculated with *Leptolyngbya* cells, adjacent to the mica flakes (Fig 1A). The samples were placed on wire racks under the same growth light as for the initial culturing (Section 2.3 above) and left under ambient conditions for two weeks. Because of the difficulty of measuring optical density when the cells were inside mica sheets, cell growth was monitored, though not quantified, using light and fluorescence microscopy as described in 2.10.

### Initial UV irradiation experiments for imaging

To evaluate *in situ* survivability, biofilms that grew into mica sheets for two weeks were exposed to UVB radiation, at a wavelength of 302 nm. UVB radiation is the most energetic region of the UV spectrum, affecting mainly cyanobacterial DNA [54]. In studies of cyanobacterial response to UVR, UVB is most commonly used [54–56]. In the initial UV irradiance experiments the petri dishes with infiltrated cells (from Section 2.4) were uncovered to prevent UV attenuation. These biofilm samples were irradiated for 0, 1, 2, 6, 12, and 24 hrs. They were subsequently observed under both a stereo and transmittance light microscope for any visual effect of UVB damage on cyanobacteria.

### Subsequent UV irradiation experiments for quantitative assays

Subsequent UV irradiation experiments required a different set up so that cell biomass could be recovered afterwards. Cells were grown for two weeks on petri dishes as outlined in 2.4 but without the mica flakes. These biofilm samples were subsequently placed underneath mica sheets of various thicknesses (approximately 50 cm x 50 cm in lateral dimension and thickness ranging from 0.18 mm to 12.93 mm for muscovite, 0.08 to 3.10 mm for biotite, and 0.09 to 11.94 mm for phlogopite) and then exposed to UVB (Fig 1B). The amount of biomass used was measured in dry weight after the UVB experiment in order to minimize any adverse effect of the drying process. The irradiance was measured via an Extech EN150 environmental multi-meter (Extech Instruments, Boston, Massachusetts) at intensity of 1147 μWcm^-2^, or 1.44 mJcm^-2^s^-1^. The amount of exposure time varied from 1 to 24 hrs. Various analyses, including microscopy, biomass analysis, pigment quantification, and phospholipid fatty acid analysis (PFLA), were carried out immediately after the irradiation experiment. All experiments were performed in duplicate with the exception of the PFLA analyses.

### Survival experiment

A separate experiment was conducted using the protection experimental setup (Fig 1B) to assess if UV-damaged cells could grow again once they were placed into a normal growth medium by following a similar method [57]. In duplicate experiments, a known amount of cells were exposed to both UVB and UVC for three time durations (1, 6, and 24 hrs), under one of the following four conditions; unprotected, protected with a muscovite sheet 0.177 mm thick, a biotite sheet and phlogopite sheet, both of which were 0.1 mm thick. Although it was ideal to keep the thickness constant for the three mica minerals, it was difficult to cleave to exactly the same thickness. For muscovite, the smallest thickness possible was 0.177 mm, but this difference in thickness did not change the result, because even with twice the thickness of phlogopite and biotite, muscovite still offered little protection. A control was grown without any UVB and UVC exposure. After UV exposure for various times, cells were placed in approximately 200 mL sterile BG-11 medium, and re-grown for three days under the normal condition (Section 2.3). The three-day re-growth period was selected because it fell within the log phase, based on the growth curve for *Leptolyngbya* sp., ATCC 29170 (S1 Fig). After re-growth, cell concentrations were measured with optical density (OD 600 nm). The percent was calculated according to the following equation:

$$Percent\;survival=\frac{M_{f}}{M_{i}}\times 100$$ Eq 1

Where M_f_ is the concentration after 3-day growth of UV-exposed cells and M_i_ is the concentration after 3-day growth of unexposed cells.

### Visible light transmittance measurements

Light transmittance through mica was measured inside a spectrophotometer (Thermo Scientific GENESYS 10S Vis) by orienting the mica flakes perpendicular to the incident beam (e.g., along the mica C-axis). Two representative wavelengths, 450 nm and 680 nm, which correspond to critical wavelengths necessary for photosynthesis [58, 59], were measured. Ultraviolet light intensity was measured using an Extech EN150 environmental multi-meter. The ability of the mica minerals in attenuating UV irradiation was measured by placing the mica between the source and the meter.

### Fe(II) and total Fe determination

Total Fe(II) concentration was measured using the standard 1,10-phenanthroline method [60]. In brief, samples were weighed and digested in 3.6 N sulfuric acid, 51% hydrofluoric acid, and 10% wt/wt 1, 10 phenanthroline in 95% ethanol until no physical sample remained. For total Fe, Fe(III) was reduced to Fe(II) using hydroxylamine hydrochloride, and the procedure was repeated.

### Imaging of cell morphology and pigment chlorophyll *a*

Cells were imaged with light microscopy using an Olympus SZX-12 stereomicroscope (Olympus Corporation, Shinjuku, Tokyo, Japan) equipped with a Nikon D300 dSLR 12 MP RBG camera and an Olympus AX-70 wide-field multi-mode microscope (Olympus Corporation, Shinjuku, Tokyo, Japan) equipped with a Nikon D300 dSLR 12 MP RBG camera and a Roper 4k cooled CCD. Chlorophyll *a* was excited using a 100 w Halogen, X-cite 120 LED lamp and filtered using bandpass filters with a Center Wavelength (CWL)/Half Bandwidth (HBW) of Full Width at Half Maximum (FWHM) 480/40 nm and emission filters at 645/75 nm.

### Quantitative measurements of pigments

Correlation between light absorption and chlorophylls has been shown in cyanobacteria [61]. The ability of the cyanobacterium to survive under high UVR exposure can be tracked by measuring the concentration of pigments necessary for the maintenance and growth of the organisms, chlorophyll *a* and carotenoids [62]. C-phycocyanin, a major light harvesting pigment, has also been shown to degrade when exposed to UVR [63, 64] and it was also measured.

Chlorophyll *a* and carotenoid concentrations were quantified following a previous protocol [65]. Briefly, irradiated samples were dried gently at 40°C, weighed, and placed in a 1.5 mL Eppendorf tube. After cooling to room temperature, 1 mL cold methanol (4°C) was added to each sample tube. The samples were agitated for one minute using a vortexer. The samples were then incubated at 4°C for 30–60 minutes. Longer incubation time would break down pigments. Following incubation, samples were centrifuged at 11,000 g for seven minutes, and the resulting pellets were checked for color. When all pigments, except phycobiliproteins, were extracted, the supernatant was then decanted into a 1 cm cuvette and measured at 470, 665, and 720 nanometer wavelengths on a Genesys 10 Vis spectrophotometer, using methanol as a blank. Because of potential interference from other compounds, three different wavelengths were used to accurately quantify chlorophyll *a* and carotenoid concentrations [65]. Specifically, the following equations were used for quantification:

$$Chl\;a\;[\mu g/ml]=12.9447\;(A_{665}-A_{720})$$

$$Carotenoid\;[\mu g/ml]=[1,000\;(A_{470}-A_{720})-2.86\;(Chla\;[\mu g/ml])]/221$$

Where Chl *a* is chlorophyll *a* concentration, and Carotenoid stands for carotenoid concentration. The concentrations of these pigments were normalized relative to dry biomass.

C-phycocyanin was estimated by treating the samples with one milliliter of 12 M HCl, vortexed, and left to digest for 24 hrs at room temperature. Optimal sample size was determined to be between 0.2 and 0.4 grams. The C-phycocyanin concentration was assayed by measuring the optical density at 652 and 620 nm using the equation from Bennett and Bogorad [66], e.g., CPC = (OD_620_-0.474 x OD_652_)/5.34. The measured concentration was normalized relative to dry biomass. This method has been shown to be more effective than typical freeze-thaw techniques, but still not yielding 100% recovery [67]. Analyses were performed in duplicate.

### Phospholipid fatty acid analysis

To measure how PLFAs respond to UVR, cyanobacterial mats were grown on plates, as previously described, and irradiated for 24 hrs underneath sheets of mica of approximately 1 mm in thickness. Two controls were included: 1) one control was not irradiated, the positive control, and the other was irradiated but without mica protection, the negative control. Lipids were extracted using a modified Bligh and Dyer method [68] in one‐phase chloroform‐methanol‐potassium hydroxide solution. Lipids were recovered, dissolved in chloroform, and fractionated on disposable silicic acid columns into neutral‐, glyco‐, and polar‐lipid fractions. The polar lipid fraction was transesterified with the mildly alkaline methanol potassium solution to recover the PLFAs as methyl esters in hexane. The polar lipid fatty acids were derivatized to fatty acid methyl esters, which were quantified by gas chromatography [69]. PLFAs were analyzed by gas chromatography (HP 5972, Agilent, Santa Clara, CA, United States) using helium as the carrier gas, with peak confirmation performed by electron ionization mass spectrometry. PLFA analyses were performed by Microbial Insights (Knoxville, Tennessee).

## Results

### Fe content in mica and their light transmission property

**Fig 2:**
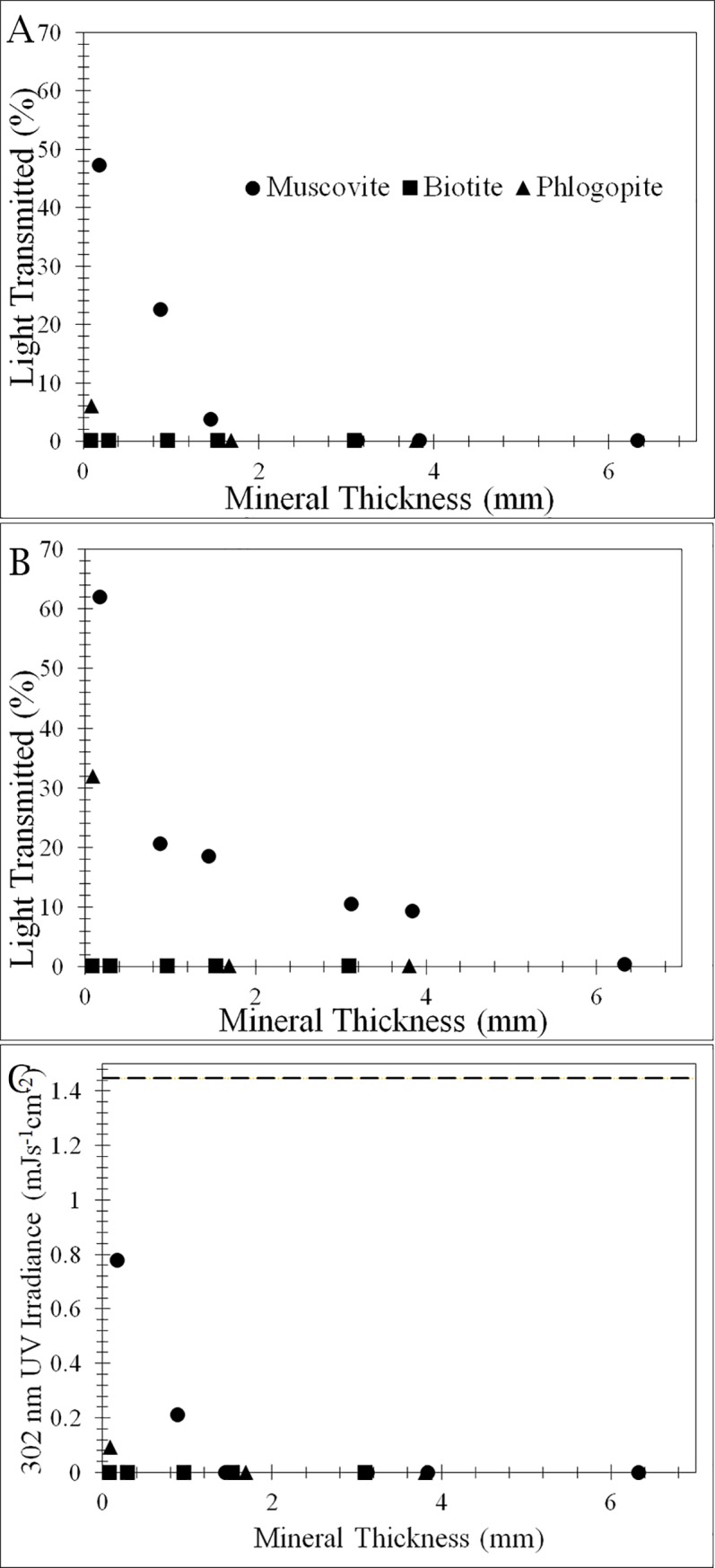
Light penetration of natural mica samples. Transmission of two photosynthetically active radiations (680 nm in A and 450 nm in B) and UVB (302 nm) as a function of mica thickness. Visible light is expressed in % transmission. UVB (302 nm) is expressed as UV light irradiance (C).

The Fe(II) concentration was 1.5%±0.03%, 2.9%±0.06%, and 12.0%±0.2% for muscovite, phlogopite, and biotite, respectively. The Fe(III) content was 0.1%±0.002%, 0.3%±0.01%, and 3.1%±0.06, respectively. Consistent with their Fe content, visible lights were transmitted through muscovite over a few millimeters in thickness (Fig 2), but the biotite and phlogopite transmittance of visible light dropped to less than 0.1% of the incident light at a thickness of < 1 mm. Light of shorter wavelength (450 nm) was able to penetrate thicker muscovite than the longer wavelength (680 nm). UVB only managed to penetrate a short distance into muscovite and phlogopite minerals (~0.8 and 0.1 mm, respectively) but was unable to penetrate through the thinnest biotite (0.1 mm). A similar trend was observed for transmittance of the entire range of UV-visible light, e.g., muscovite was the most transmissive, biotite the least (S2 Fig).

### Cell colonization into mica sheets and survival upon UVB exposure

**Fig 3:**
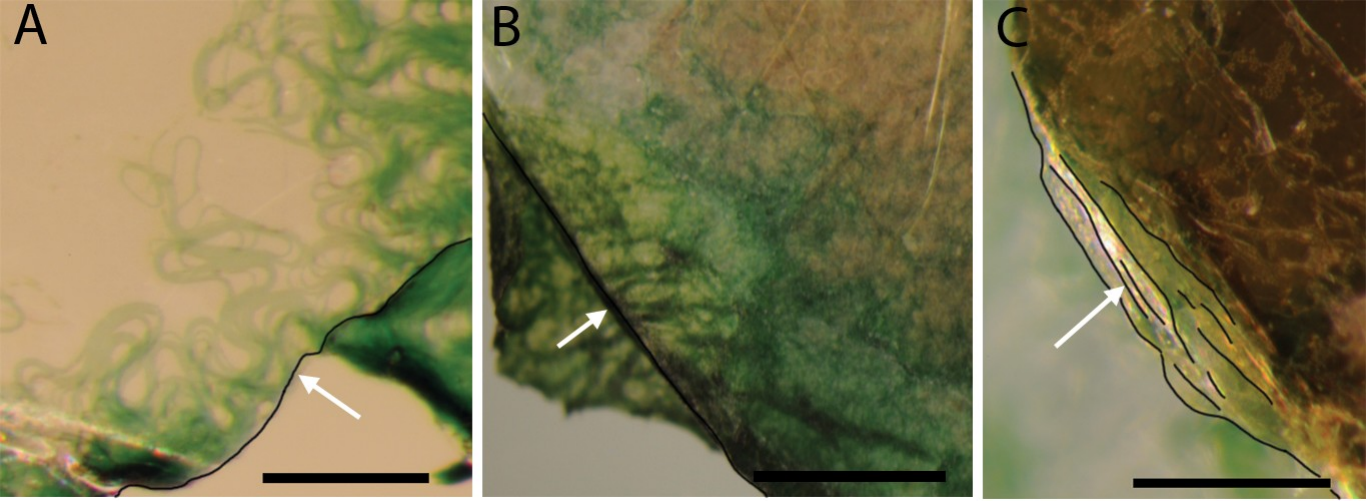
Stereo microscope images showing infiltration of filamentous cyanobacteria into various mica sheets after two weeks of growth. Images were taken looking down the c-axis, per experimental setup (Fig 1A). The edges of mineral sheets are highlighted with black curves. (A) shows infiltration of green cyanobacterial cells into sheets of muscovite (the direction of infiltration is from the lower right corner of the image to the upper left, as indicated by the white arrow. Scale bar is 100 microns; (B) shows infiltration into phlogopite (from the lower left to the upper right direction, as indicated by the white arrow). Scale bar is 50 microns; and (C) shows growth into multiple sheets of biotite (from the lower left to the upper right direction). Scale bar is 50 microns.

Light microscopy examination revealed that cyanobacterial filaments were able to colonize and grow between mica sheets. Individual filaments of cyanobacteria were able to penetrate over 100 microns into the sheets of muscovite and phlogopite (Fig 3A and 3B). Biotite with broken stepwise edges was also colonized, but the penetration distance appeared to be much shorter (Fig 3C), possibly because it was difficult to observe the biofilm underneath opaque biotite sheets.

**Fig 4:**
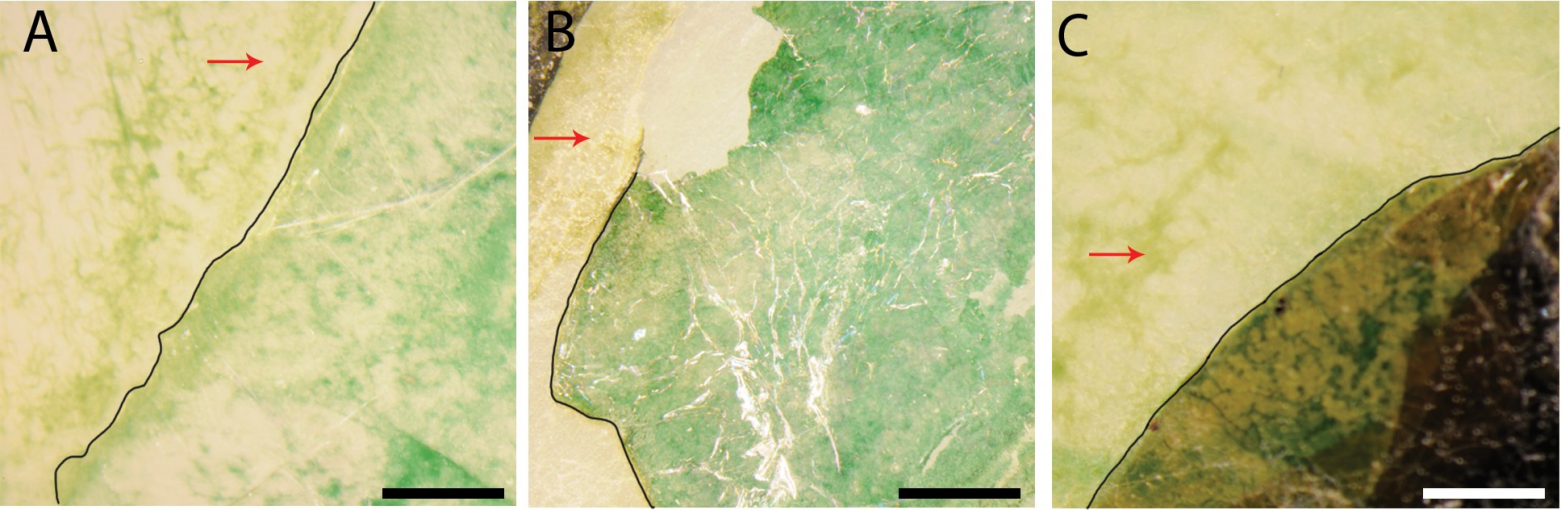
Stereo microscope images showing discoloration of damaged cells after exposure of UVB at 302 nm. Partially infiltrated cyanobacterial biofilms into muscovite (A), phlogopite (B), and biotite (C) sheets were exposed to UVB for 6 hours. The edges of mineral sheets are highlighted with black curves. The biofilms protected underneath mica sheets (the lower right portion of the images) remained green in color, while those regions without any mica protection (the upper left regions, indicated by red arrows) showed discoloration. Images were taken looking down the C-axis. Each scale bar represents 50 microns.

Visual observation of UVB-exposed cells revealed that the cells under mica protection did not become discolored after exposure to UVB (the bluish-green regions in the lower right portion of the images on Fig 4), but without mica protection, cells showed discoloration (the yellow regions in the upper left region in Fig 4, indicated by red arrows). The discoloration from bluish-green to yellow suggests a loss in green pigments, likely chlorophyll and phycocyanobilin, while the green color persists in cells found underneath mica layers.

**Fig 5:**
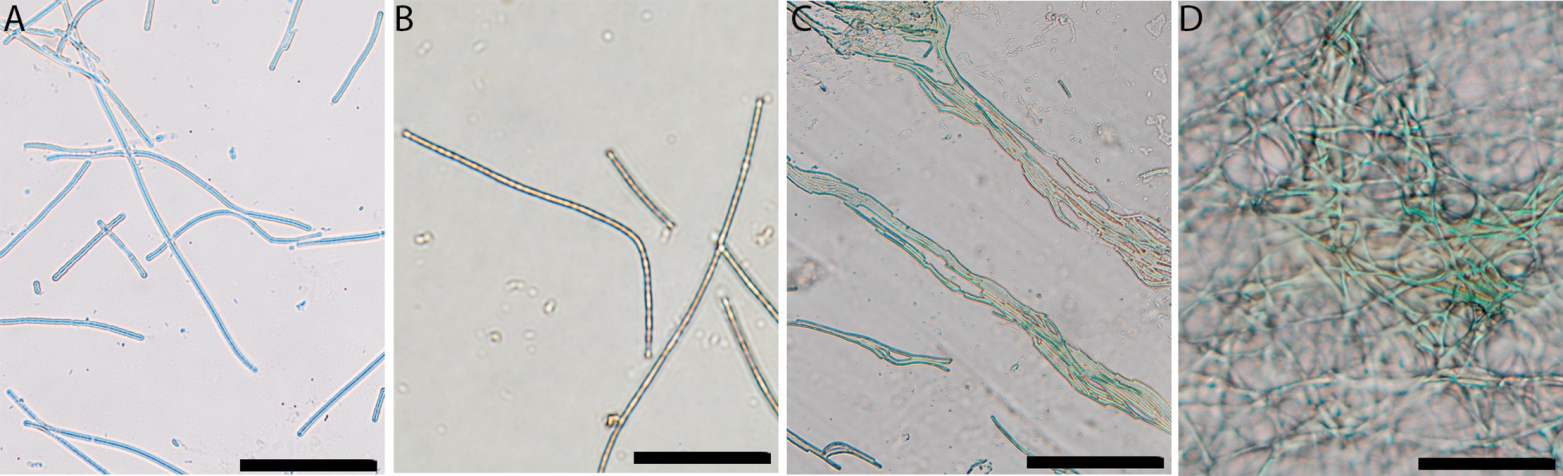
Transmitted light image of photobleached cells. Transmitted light image of cells. (A) Completely photo-bleached cells under 1.45 mm thick muscovite with no remaining chlorophyll *a* and carotenoids after 24 hr UVB exposure. The blue color is from phycocyanin. (B) Photo-bleached cells (mostly orange coloration) under 1.67 mm thick phlogopite with carotenoids mostly remaining after 24 hr UVB exposure. (C) Slightly photo-bleached cells (green-blue with some orange color) under 1.53 mm thick biotite with most chlorophyll *a* and carotenoids still remaining after 24 hr UVB exposure. (D) Healthy cells without any UVB exposure showing green color. Scale bar is 50 microns.

To further reveal the reasons for the observed discoloration, transmitted and fluorescent images were obtained. When the cells inside muscovite sheets were exposed to UVB, all chlorophylls and carotenoids were destroyed in these cells, leaving behind only the faint blue color characteristic of phycocyanin (Fig 5A). In contrast, when the cells were under either phlogopite or biotite sheets, they were better protected, as shown by a mixture of green pigments from chlorophyll *a* and an orange-yellow hue from the carotenoids (Fig 5B and 5C). Healthy cells (not exposed to UVB) showed a characteristic green color (5D).

**Fig 6:**
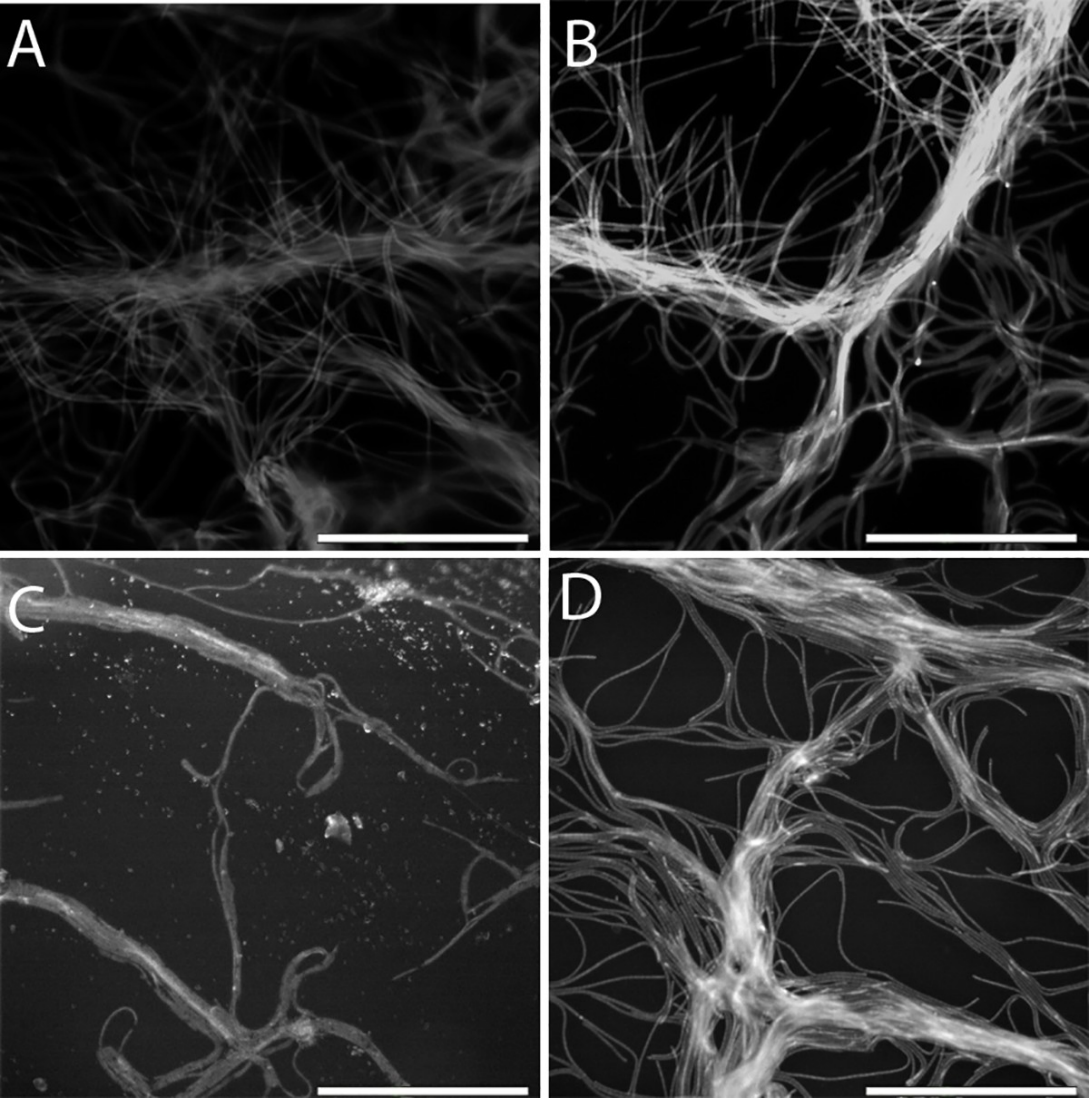
Fluorescent images showing UVB-induced chlorophyll *a* degradation as a function of mineral type and thickness. Under thin sheets of phlogopite and biotite (A and C, respectively, both at 0.1 mm), chlorophyll *a* was partially destroyed, as indicated by the low fluorescent intensity of the individual filaments. Under thicker phlogopite and biotite sheets (B at 3.1 mm and D at 3.8 mm, respectively), chlorophyll *a* was largely preserved as indicated by the higher signal intensity. Individual cells are clearly visible. There is little difference in chlorophyll *a* degradation between phlogopite (A and B) and biotite (C and D). For muscovite, even at a small thickness, chlorophyll *a* was completely photo-bleached (images not shown).

Similar patterns of the protective role of the micas were revealed by fluorescent images. While muscovite had no protective role (data not shown), phlogopite and biotite sheets offered protection, with the level of protection depending on the thickness (Fig 6). At 0.1 mm thickness, chlorophyll *a* was partially destroyed (Fig 6A and 6C for phlogopite and biotite, respectively), but a greater thickness (3.1–3.8 mm) almost completely protected chlorophyll *a* from degradation by UVB (Fig 6B and 6D).

**Fig 7:**
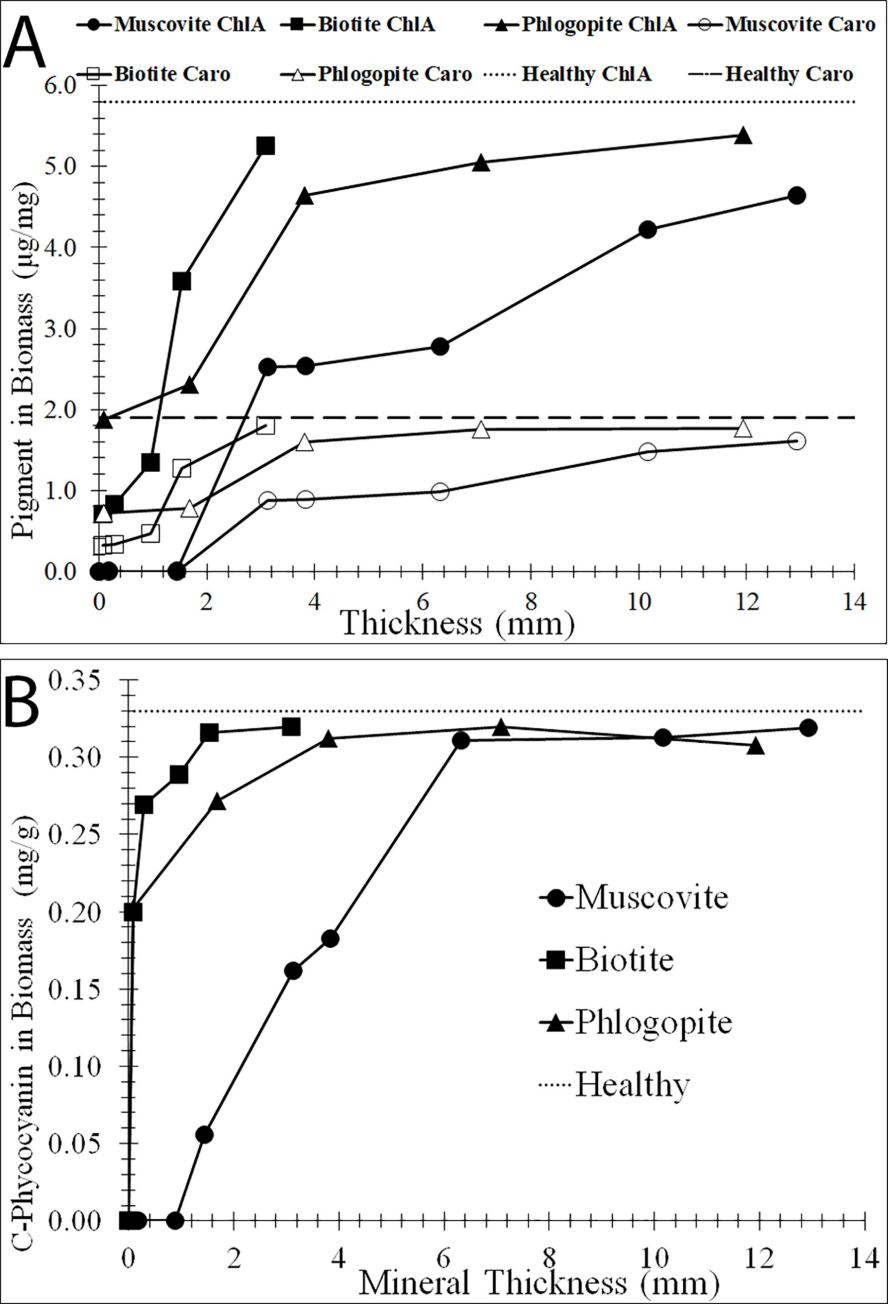
Change of cyanobacterial pigments as a function of mica thickness. Concentrations of chlorophyll *a*/carotenoids (A) and C-Phycocyanin (B) as a function of mica thickness, measured after exposure to UVB irradiation at 302 nm for 24 hrs. Concentrations of pigments were normalized to dry biomass. Positive controls, exposed to UVB for 24 hrs without any mineral protection, yielded no measurable levels of chlorophyll *a* or carotenoids (data not plotted). Zero thickness corresponds to no-mineral protection. Pigment concentrations of healthy cells (no UVB exposure) are plotted as dotted or dashed lines.

The level of protection of the cyanobacterium by the mica minerals was further quantified in terms of pigment decay as a function of mica thickness (Fig 7). Without any mica protection (e.g., 0 thickness in Fig 7), all three pigments, chlorophyll *a*, carotenoids, and C-Phycocyanin, significantly decreased in abundance per mg of biomass relative to healthy cells. In the presence of the mica minerals, these pigments were protected to various extents. As expected, for all three minerals, chlorophyll *a* and carotenoids were better protected as the thicknesses of the micas increased (Fig 7A). Among the three mica minerals, muscovite was the least effective in protecting the cyanobacterium from irradiation, as shown by its lowest levels of chlorophyll *a* and carotenoid when compared to other micas of similar thickness. When the muscovite thickness was < 2 mm, no chlorophyll *a* or carotenoid was detected, suggesting that these pigments were completely destroyed by UVB. In contrast, because the phlogopite and biotite substantially attenuated UVB even when the sheets were less than a millimeter in thickness (Fig 2C), chlorophyll *a* and carotenoids were better preserved by these minerals when the cells were exposed to the same UVB (Fig 7A). Relative to chlorophyll *a* and the carotenoids, C-Phycocyanin was more resistant to UVB, as evidenced by its nearly complete protection by thin sheets of phlogopite and biotite.

### Cell survival after UVB and UVC exposure

**Fig 8:**
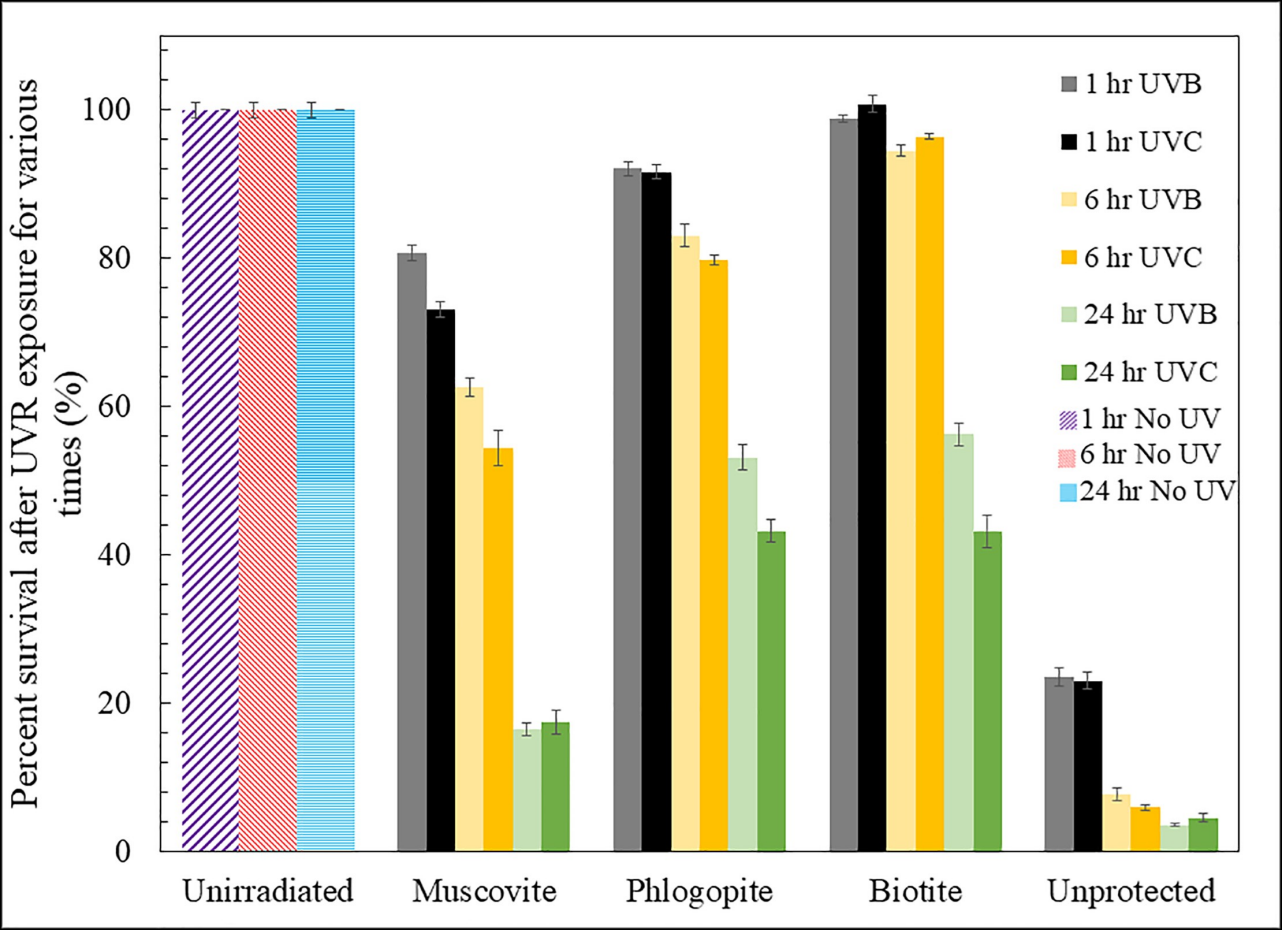
Biomass survival as a function of cell exposure duration, UV wave length, and the mineral type. After three days of re-growth, cyanobacterial populations survived better when they were shielded by biotite and phlogopite than by muscovite, even though the muscovite thickness (0.177 mm) was greater than those of phlogopite and biotite (both at ~0.1 mm). Relative to UVB, exposure to UVC resulted in a lower survival rate. Likewise, longer exposure resulted in a lower survival rate. Unprotected cells were nearly completely killed in 24 hrs. Error bars indicate standard deviation from experimental duplicates.

To determine if UVR-exposed cells were able to survive and recover once UVR stress was removed and cells were inoculated into normal BG-11 medium, cell growth, relative to unirradiated cells, was tracked after a three-day recovery period (Fig 8). The percent survival of irradiated cells depended on the duration of irradiation, wavelength (e.g., UVB or UVC), and the mineral type. As expected, across all treatments, a longer duration of irradiation resulted in a lower fraction of survival. Exposure to UVC irradiation resulted in a lower survival than exposure to UVB. Relative to unprotected cells, mica minerals offered protection to varying extents Among the three mica minerals, muscovite was the least effective, as evidenced by the lowest cell survival, relative to biotite and phlogopite, even when the muscovite (0.177 mm) was thicker than those of phlogopite and biotite (both 0.1 mm).

### Change of PLFA profile upon UVR exposure

**Fig 9:**
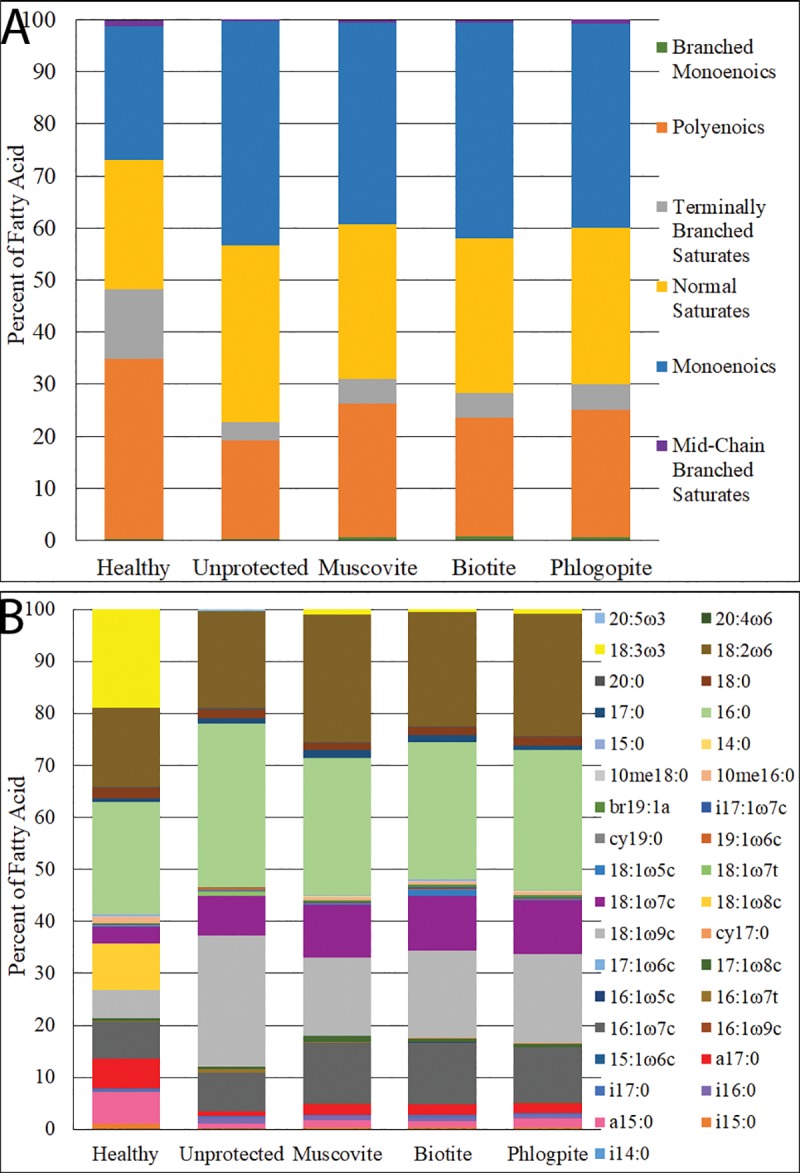
Alterations in fatty acid compositions of *Leptolyngbya* populations after exposure to UVB for 24 hrs. Classes of acids are shown in (A), while individual fatty acids are shown in (B). These shifts are indicative of cellular changes due to UVB exposure, with the largest change shown between the healthy and unprotected cells. The thickness of all three mica minerals was ~0.1 mm.

To further determine if cell membrane composition responds to UVR, PLFA was analyzed for the cells that were exposed to UVB for 24 hrs. The total amount of PLFA decreased from 165.1 nmol/g (biomass) for healthy cells to 40.3 nmol/g after 24 hours of UVB exposure. In the presence of 0.1 mm mica minerals, the total amount of PLFA remained nearly at the same level as healthy cells, with 169.6, 146.7, and 156.4 nmol/g for muscovite, phlogopite, and biotite, respectively. However, there were dramatic changes in the composition of PLFAs (Fig 9A), suggesting cellular changes due to the stress of UVB. Relative to the healthy cells, UVB exposure diminished terminally branched saturates, decreased the content of polyenoics, but increased the content of monoenoics.

At the individual fatty acid level, only a few fatty acids displayed major changes (Fig 9B). Specifically, the proportion of the α-linolenic acid (bright yellow color in Fig 9B), abbreviated as 18:3ω3, diminished to nil from healthy to unprotected cells, with only minor percentages left in the mica-protected cells. The 8-octadecenoic acid (golden color), abbreviated 18:1ω8c, was not detected in both unprotected and mica-protected cells either. a15:0 (the pink color) and a17:0 (the red color) both showed a marked decrease when cells were unprotected, but minor amounts persisted in mica-protected cells. In contrast, there were significant increases in 18:1ω9c (the light grey color) and 18:1ω7c (the purple) after UVB exposure. The abundance of 18:1ω9c showed the greatest increase, from 5% to 25% after irradiation, even in mica-protected cells. The three mica-protected samples showed a similar percentage increase of this acid, at ~15%.

## Discussion

### Mechanisms of cyanobacterial response to UVR

Previous studies have shown decay of chlorophyll *a* and C-Phycocyanin upon exposure of various cyanobacteria to UVB [70]. Decrease in chlorophyll *a* content has been associated with inhibition of aminolevulinic acid synthesis [71] or a reduction in protochlorophyllides [55]. The destruction of chlorophyll *a* by UVR comes with a comparative loss of photosynthetic capacity. The observed decrease in chlorophyll *a* concentration when exposed to UVB suggests that the chlorophyll synthesis machinery may have been damaged or repressed under UV-B exposure. Thus, loss of pigments is likely due to not only the destruction of available chlorophyll *a* but also a decreased level of production through DNA damage [72].

Ultraviolet irradiation stress on cyanobacteria has long been known to increase reactive oxygen species (ROS) generation [69, 73, 74]. Upon UVR exposure, the redox activity of photosynthetic pigments such as chlorophyll and phycobiliproteins, may transform the oxygen molecules into superoxide radicals, hydroxyl radicals, hydrogen peroxide, and singlet oxygen [75]. These ROS have been shown to cause oxidative stress, lipid peroxidation, DNA breaks, decreased photosynthetic function, and chlorophyll bleaching [76–78]. Therefore, scavenging of ROS would alleviate some of these damages caused by UVR exposure. Carotenoids possess this capacity of scavenging ROS [76]. Therefore, the decreased carotenoid content upon UVR sorption may be a result of scavenging ROS, as consistent with previous studies [3, 79]. However, other studies have also observed increased production of carotenoids in cyanobacteria upon UVB irradiation [4, 80], again due to their antioxidant functions. Thus, it appears that if/when there are sufficient carotenoids available to remove ROS, these compounds may be used to quench ROS and in doing so, these compounds are destroyed. However, if no sufficient carotenoids are available, UVR may induce initial production of carotenoids followed by destruction, as observed by a previous study, where carotenoid content initially increased but subsequently decreased upon prolonged UVR exposure [79].

In addition to the pigment changes, fatty acid profiles of cyanobacteria may be altered when exposed to environmental stress [16, 81, 82]. While some site alteration of fatty acids by exposure to UVR has been described previously [82], to our knowledge there is limited study on the effect of UVR on PLFA composition of cyanobacteria. One study [83] examined changes in the lipid bilayer upon exposure to UVR-induced ROS and showed that unsaturated lipids deteriorated faster than saturated, due to the lower packing density of unsaturated lipids, which would increase the mobility of ROS. The fatty acid alkyl chains are oxidized at double bond sites as well as any adjacent allylic carbons. Thus, any unsaturated fatty acids would be susceptible to degradation via photooxidation.

The complete removal of the α-linolenic acid and 8-octadecenoic after UVR exposure, two unsaturated fatty acids observed in this study, suggests a strong response of cyanobacterial phospholipid fatty acids to UVR. In response to environmental and other changes, cells can produce and regulate jasmonic acid, which is ultimately derived from linolenic acid via the octadecanoid pathway [84]. Additionally, linoleate–the salt or estered version of the acid, reacts with ROS nearly forty times faster than oleate or other higher order polyenes to form free radicals [85], which may further explain the marked decrease in α-linoleic acid (Fig 9). The drastic increase in oleic acid (18:1ω9c, Fig 9) observed in this study could be a biomolecular-level physiological response to UVR. Because oleic acid is a precursor molecule of α-linoleic acids, the increase in production of oleic acid may be a result of replenishing α-linoleic acid levels [86]. Indeed, recent research suggests that oleic acid is capable of diminishing the effects of ROS [87] that may be produced upon UVR exposure of cyanobacterial cells. In the presence of O_2_, UV irradiation can produce O_3_, which may cause oxidative damage as well. Therefore, the observed changes in lipids and fatty acids may be a consequence of oxidative damage as well, which deserves further study.

Despite these UVR-induced damage, our survival experiments showed that *Leptolyngbya* cells were able to survive and completely recover if the cells were protected underneath biotite and if the duration of UVR exposure was short (within an hour, Fig 8). With prolonged exposure, cells gradually lost their ability to revive, even in the presence of phlogopite and biotite (Fig 8). Previous studies have proposed several mechanisms to restore damaged DNA, including photoreactivation, nucleotide excision repair, and recombinational repair [5], but the relative importance of these mechanisms deserves further study, especially in the presence of mineral protection.

### Factors controlling UVR protection by mica minerals

Our results show that the three mica minerals exhibited a distinctly different behavior in attenuating UVR and protecting the cyanobacterium. Because these three minerals are iso-structural, the difference in their UVR attenuating properties should be related to the chemical composition and thus optical properties. Biotite, being an Fe(II) end member, is most effective, while muscovite, being an Al-rich end member, is the least effective. Phlogopite, being a Mg end member, partially blocks UVB (Fig 2) but allows adequate transmission of visible light. Biotite contains the most amount of iron, which increases its density and refractive index. Muscovite contains the least amount of iron and therefore has the lowest density and refractive index. Phlogopite is in the middle in terms of Fe content and would transmit more visible light than biotite but still attenuate an adequate amount of UVR. This effect of Fe on UVR attenuation and microbial protection is broadly consistent with recent studies on the role of Fe on UV attenuation [41, 42], which showed the importance of Fe(III) on UV attenuation. In our study, both Fe(II) and Fe(III) contents increased from muscovite to phlogopite to biotite, thus, it remains unclear what form of Fe, Fe(II) or Fe(III) is more important in UV attenuation. Equally unclear is whether or not Fe(II) or Fe(III) selectively attenuates certain UV irradiation.

Based on a combination of PAR transmittance and UV attenuation, we conclude that phlogopite is the superior mineral in terms of survival and growth, likely due to an intermediate quantity of Fe in the structure. Additionally, there are other iso-structural minerals with varying chemistries, which may also prove somewhat suitable environments for cyanobacteria, with paragonite being the most attractive as it contains higher levels of both iron and magnesium, however it is far less common than muscovite, phlogopite, and biotite. Future experiments should examine other Fe-bearing minerals and the effects of other factors, such as Fe oxidation state and structural defect density on the protective role of minerals against UVR.

### Implications for life-mineral co-evolution on early Earth and elsewhere

The findings of this study are of interest when considering the life-mineral co-evolution on early Earth. The phyllosilicates used in this study may have originated and persisted since the initiation of igneous rock evolution, between 4.55 and 4.0 Ga [88]. The earliest rocks with signs of life are the Isua Greenbelt formation, which points to the existence of cyanobacteria as long ago as 3.7 Ga [89], indicating that phyllosilicates would have been readily available to protect such organisms when they emerged under intense UV irradiation. Recent studies [41, 42] have shown the importance of solid-form Fe(III) (in the forms of Fe oxides or Fe(III)-Si precipitates) in shielding UVR, but these oxidized forms of Fe are unlikely abundant in a reducing early earth environment. Instead, Fe(II) minerals would be more abundant. Our data suggest that Fe(II) is important in shielding UVR as well. A recent study [42] observed that short periods of UVC exposure, even in the presence of Fe(III)-Si precipitates, can still result in high mortality rates of cyanobacteria, suggesting that other factors, such as Fe(II)-bearing minerals, may be important in life protection and proliferation on early earth. Indeed, our data demonstrated that the three phyllosilicates used in this study are protective against both UVB and UVC (Fig 8).

The significance of the micas providing potential habitats may not be unique to early earth. Both biotite and phlogopite have been found in samples of Martian meteorites [90], on Mars [91–93] and terrestrial analogs to Mars (e.g., deserts) [46–48]. R chondrites have even been shown to contain pre-terrestrial hydroxyl bearing minerals, including the micas [94]. Even with a high influx of UVR on the Martian and desert surface, microbial life, including cyanobacteria, could find refuge under these protective minerals. These small-scale habitats could have allowed for the development of photosynthetic processes from abundant carbon dioxide [95] and water in hydrous minerals [91, 96]. Indeed, both lichen and cyanobacteria from harsh terrestrial environments, such as the Alps and polar regions, were able to survive in the simulated Martian atmosphere as well as under low pressure and high UV radiation, suggesting a potential for their survival on the Martian surface [97–99]. Likewise, the habitability potential of phyllosilicates and iron-rich environments have been proposed for terrestrial analogs of Mars [46]. Although the biomolecular adaptations are necessary for these organisms to survive in such harsh conditions, physical protection, such as small-scale mineral habitats, may allow them to survive longer. Therefore, the potential for increased survivability in the presence of Fe-bearing minerals should be investigated further in the future.

## Supporting information

S1 Fig Growth curve for *Leptolyngbya* in BG-11 medium. (DOCX)

S2 Fig Transmittance of UVR and PAR as a function of wavelength. (DOCX)
