# Supplementary material for: Phyllosilicates as protective habitats of filamentous cyanobacteria Leptolyngbya against ultraviolet radiation
Source: PLoS One. 2019 Jul 11;14(7):e0219616. doi: 10.1371/journal.pone.0219616 (PMC6623962; doi:10.1371/journal.pone.0219616)
Supplement: S1 Fig — (DOCX) [file pone.0219616.s001.docx]

**Phyllosilicates as protective habitats of filamentous cyanobacteria *Leptolyngbya* against ultraviolet radiation**

**Micas as potential UV-resilient habitats for cyanobacteria**

^1^Alex Kugler and ^1^Hailiang Dong

^1^Department of Geology and Environmental Earth Sciences, Miami University, Oxford, OH 45056, USA

Corresponding author: Hailiang Dong

Department of Geology & Environmental Earth Sciences

Miami University, Oxford, Ohio, USA

Tel: 513 529 2517

Fax: 513 529 1542

Email: dongh@miamioh.edu

Further Revised for PLOS ONE

June 25, 2019

**S1 Fig. Growth curve for *Leptolyngbya* in BG-11 medium.** Cells were grown under the conditions described in Section 2.3. Optical density at 600 nm (OD_600_) was used for biomass measurement. Error bars are smaller than the size of symbols.
