# Supplementary material for: Phyllosilicates as protective habitats of filamentous cyanobacteria Leptolyngbya against ultraviolet radiation
Source: PLoS One. 2019 Jul 11;14(7):e0219616. doi: 10.1371/journal.pone.0219616 (PMC6623962; doi:10.1371/journal.pone.0219616)
Supplement: S2 Fig — (DOCX) [file pone.0219616.s002.docx]

**Phyllosilicates as protective habitats of filamentous cyanobacteria *Leptolyngbya* against ultraviolet radiation**

**Micas as potential UV-resilient habitats for cyanobacteria**

^1^Alex Kugler and ^1^Hailiang Dong

^1^Department of Geology and Environmental Earth Sciences, Miami University, Oxford, OH 45056, USA

Corresponding author: Hailiang Dong

Department of Geology & Environmental Earth Sciences

Miami University, Oxford, Ohio, USA

Tel: 513 529 2517

Fax: 513 529 1542

Email: dongh@miamioh.edu

Further Revised for PLOS ONE

July 1, 2019


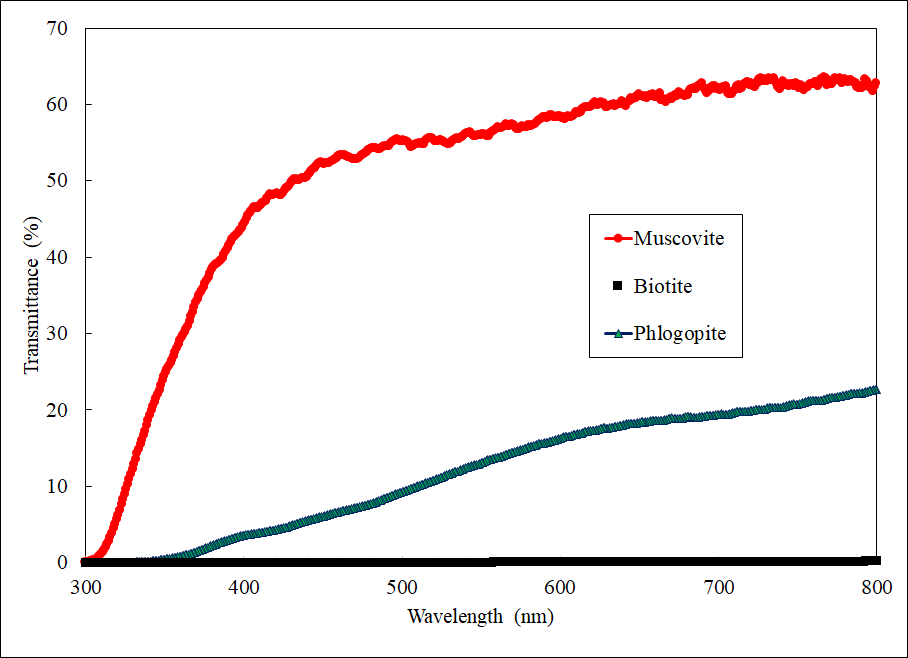


**S2 Fig. Transmittance of UVR and PAR as a function of wavelength.**Transmission percentage of UVR and PAR ranging from 300 to 800 nm is shown for three mica minerals. Each scan was performed on the thinnest piece of mica used in Figures 2 and 7. The muscovite used was 0.177 mm, the biotite 0.083 mm, and the phlogopite 0.091 mm thick. Because these are natural samples, it is difficult to keep exactly the same thickness. However, despite that muscovite is thicker than phlogopite and biotite, its transmission is still the highest. Biotite shows the least transmittance. These samples were placed perpendicular to the incident beam and the percent transmission of light recorded.
